# Vitamin D status and Its association with haematological inflammatory indices and cardiometabolic risk profiles: a retrospective cross-sectional study

**DOI:** 10.3389/fcvm.2026.1859225

**Published:** 2026-06-10

**Authors:** Yaqeen Mohammed Al-Essa, Hussain Khalifa Aljumah, Zainab Younis Al saeed, Raneem Abdullah AlQattan, Danah Yousef Alquwayzani, Ghadeer kazem Al-Yousef, Ayah Salem AL owdah, Mujtaba Abbas Alzuwayr, Eman Elsheikh

**Affiliations:** 1 King Faisal University College of Medicine, Al Ahsa, Saudi Arabia; 2 Internal Medicine Department, College of Medicine, King Faisal University, Al-Ahsa, Saudi Arabia; 3 Cardiology Department, Faculty of Medicine, Tanta University Hospital, Tanta, Egypt

**Keywords:** cardiometabolic risk, Haematological inflammatory indices, Neutrophil-to-lymphocyte ratio, retrospective cross-sectional study, systemic immune-inflammation index, vitamin D deficiency

## Abstract

**Background:**

Cardiovascular disease remains the leading cause of global mortality, with dyslipidaemia and impaired glycaemic control representing central modifiable risk factors. Haematological inflammatory indices and vitamin D status have each been independently associated with cardiovascular risk; however, their potential interaction remains underexplored. This study evaluated whether vitamin D status exhibited differential relationships with cardiometabolic risk markers.

**Methods:**

This retrospective cross-sectional study included 1,100 adults attending King Faisal University polyclinics between January and March 2026. Participants were categorised as vitamin D sufficient (≥30 ng/mL, *n* = 175), vitamin D insufficient (20–29 ng/mL, *n* = 470), and vitamin D deficient (<20 ng/mL, *n* = 455). Haematological inflammatory indices, including the neutrophil-to-lymphocyte ratio (NLR), platelet-to-lymphocyte ratio (PLR), monocyte-to-lymphocyte ratio (MLR), and systemic immune-inflammation index (SII), were calculated. Associations with lipid and glycemic parameters were evaluated using correlation analyses, logistic regression, and receiver operating characteristic curve analysis.

**Conclusions:**

Vitamin D deficiency was associated with adverse inflammatory and cardiometabolic profiles, indicating that low vitamin D status coexisted with less favorable inflammatory and metabolic profiles. Although the NLR, PLR, and SII were consistent markers, their modest discriminatory performance limits standalone diagnostic use. These findings suggest that vitamin D status may be associated with differential relationships between haematological inflammatory indices and cardiometabolic risk markers.

## 1 Introduction

Cardiovascular disease (CVD) remains a leading cause of morbidity and mortality worldwide (1, 2) and is strongly correlated with metabolic risk factors, such as dyslipidaemia and impaired glycaemic control (3, 4). Chronic low-grade inflammation has also been consistently associated with the development and progression of cardiometabolic disease (5–11).

Haematological inflammatory indices based on routine complete blood count parameters, including the neutrophil-to-lymphocyte ratio (NLR), platelet-to-lymphocyte ratio (PLR), monocyte-to-lymphocyte ratio (MLR), and systemic immune-inflammation index (SII), are markers of systemic inflammation and cardiometabolic risk (12–18). Similarly, vitamin D deficiency has been associated with adverse inflammatory and metabolic profiles in epidemiological studies (19–23). Although both vitamin D status and haematological inflammatory indices have been individually associated with cardiometabolic risk, these factors are often evaluated in isolation in observational studies (24, 25). In many studies, vitamin D is treated as a covariate rather than examined in terms of differences in inflammation-associated metabolic risk (20, 21). Thus, there are limited data on potential differences in the associations between haematological inflammatory indices and cardiometabolic parameters by vitamin D status.

This study was conducted to explore the associations of vitamin D status with haematological inflammatory indices and cardiometabolic risk markers in adults attending a university polyclinic. In particular, we explored whether associations between inflammatory indices and metabolic parameters differed across vitamin D categories.

## 2 Methods

### 2.1 Study design and setting

This retrospective cross-sectional analytical study was conducted to evaluate associations among vitamin D status, haematological inflammatory indices, and cardiometabolic parameters, including lipid and glycaemic profiles. Data were obtained from the electronic medical records of patients attending King Faisal University (KFU) polyclinics from January 1, 2026, to March 1, 2026.

### 2.2 Study population

Adult patients ≥ 18 years with complete laboratory data, including serum vitamin D levels, complete blood counts (CBCs), lipid profile, and glycaemic parameters, were considered eligible for inclusion. Patients were excluded if they had any of the following conditions: acute infections or inflammatory conditions at the time of sampling; chronic inflammatory or autoimmune diseases; malignancies; haematological disorders; chronic liver or kidney disease; or current use of corticosteroids or immunosuppressive therapy. Additionally, records with incomplete or missing key variables were excluded.

After applying the eligibility criteria, 1,100 patients were included in the final analysis.

### 2.3 Sample size and patient selection

During the study period, approximately 5,000 patients attended KFU polyclinics. Of these, 2,200 were excluded according to the predefined criteria (e.g., age < 18 years, the presence of infections or chronic diseases, or incomplete laboratory investigations required for the study variables). A total of 2,800 patients were initially selected for data collection. Subsequently, 150 records were excluded due to missing data, resulting in 1,150 eligible patients. Finally, 1,100 patients were included in the analysis.

### 2.4 Data collection

Demographic and clinical data, including age, sex, body mass index (BMI), and comorbidities, such as diabetes mellitus and hypertension, were extracted using a standardised data collection form. Laboratory parameters were retrieved from the institutional laboratory database.

### 2.5 Laboratory measurements

#### 2.5.1 Haematological parameters

Haematological parameters, including total white blood cell, neutrophil, lymphocyte, and platelet counts, were measured using an automated haematology analyser (Sysmex XN-1000, Sysmex Corporation, Japan) based on fluorescence flow cytometry and impedance principles. Derived haematological inflammatory indices were subsequently calculated, including the NLR and PLR.

#### 2.5.2 Biochemical analysis

Biochemical analyses were performed using the VITROS 7600 automated chemistry analyser (Ortho Clinical Diagnostics, USA), employing standardised enzymatic and immunodiagnostic techniques.

#### 2.5.3 Glycaemic parameters

Fasting blood glucose (FBG) was measured using an enzymatic method based on glucose oxidase or glucose dehydrogenase reactions. In this process, glucose undergoes enzymatic oxidation, producing hydrogen peroxide, which generates a measurable colorimetric change proportional to the glucose concentration.

Glycated haemoglobin (HbA1c) was determined using an immunoassay technique. Haemoglobin binds to specific antibodies targeting glycated haemoglobin, enabling the quantification of HbA1c as a percentage of total haemoglobin, reflecting average blood glucose levels over the preceding 2–3 months.

#### 2.5.4 Lipid profile

The lipid profile, including total cholesterol, triglycerides, high-density lipoprotein cholesterol, and low-density lipoprotein cholesterol, was assessed using enzymatic colorimetric assays. Total cholesterol and triglycerides were measured directly, whereas HDL cholesterol was determined following selective inhibition or precipitation of non-HDL lipoproteins. LDL cholesterol was either calculated using the Friedewald equation or directly measured, depending on triglyceride levels.

#### 2.5.5 Vitamin D measurement

Serum 25-hydroxyvitamin D concentrations were measured using a chemiluminescent immunoassay. In this competitive assay, circulating vitamin D competes with labelled vitamin D for antibody binding, and the emitted signal is inversely proportional to the vitamin D concentration. Vitamin D status was classified as vitamin D deficiency (<20 ng/mL), vitamin D insufficiency (20–29 ng/mL), or vitamin D sufficiency (≥30 ng/mL). For the primary analysis, patients were also categorised into vitamin D-deficient, vitamin D-insufficient, and vitamin D-sufficient groups.

### 2.6 Statistical analysis

Statistical analysis was performed using SPSS version 28 (IBM©, Armonk, NY, USA). Data normality was assessed using the Shapiro–Wilk test and a histogram.

Quantitative parametric data are expressed as the mea*n* ± standard deviation (SD) and were analysed using one-way analysis of variance (ANOVA) with Tukey’s *post hoc* test. Non-parametric data are presented as median and interquartile range (IQR) and were analysed using the Kruskal–Wallis test followed by Mann–Whitney U tests for pairwise comparisons. The Bonferroni test (or correction), a statistical method used to reduce Type I errors (false positives), was employed when conducting multiple, simultaneous comparisons.

Categorical variables are expressed as frequencies and percentages and were analysed using the Chi-square test. A two-tailed *p* value < 0.05 was considered to indicate significance. Given the exploratory nature of the analyses, *p* values were interpreted with caution.

Correlation analysis was performed using Pearson or Spearman correlation coefficients to evaluate associations between quantitative variables.

Univariate logistic regression analyses were performed to evaluate associations between inflammatory and metabolic parameters and vitamin D status. Significant variables were included in the multivariable logistic regression analyses. Odds ratios (ORs), 95% confidence intervals (CIs), and *p* values were reported.

Multivariable logistic regression models were additionally constructed to evaluate interaction terms between vitamin D status and haematological inflammatory indices. Vitamin D deficiency status was used as the dependent variable. Interaction terms involving NLR, PLR, MLR, and SII were entered separately into adjusted models controlling for age, sex, BMI, and smoking status.

### 2.7 Roc curve analysis

Receiver operating characteristic (ROC) curve analysis was performed to evaluate the diagnostic performance of inflammatory and metabolic parameters in predicting vitamin D status. Curves approaching the upper left corner indicated better diagnostic performance. The area under the curve (AUC) was used as a measure of test accuracy. The area under the curve (AUC) was used as a measure of discriminatory performance, where values closer to 1.0 indicate better performance, whereas values near 0.5 indicate poor discrimination.

### 2.8 Ethical considerations

All data were anonymised prior to analysis to ensure patient confidentiality. The study protocol was approved by the Institutional Review Board of King Faisal University (Ref: KFU-REC-2026-JAN–ETHICS3920). Informed consent was waived due to the study’s retrospective nature. The study was conducted in accordance with the principles of the Declaration of Helsinki.

## 3 Results

A total of 1,100 participants were categorised into three groups according to vitamin D status: vitamin D-sufficient (*n* = 175), vitamin D-insufficient (*n* = 470), and vitamin D-deficient (*n* = 455). Baseline demographic characteristics, including age, sex, BMI, and smoking status, did not differ significantly across groups (Table 1).

**Table 1:** Baseline characteristics of the studied groups.

Characteristic	Category/statistic	Group 1 (Sufficient) (*n* = 175)	Group 2 (Insufficient) (*n* = 470)	Group 3 (Deficient) (*n* = 455)	*P* value
Age (years)		48.5 ± 14.2	50 ± 14.03	48.9 ± 13.81	0.367
Sex	Male	85 (48.6%)	243 (51.7%)	231 (50.8%)	0.778
	Female	90 (51.4%)	227 (48.3%)	224 (49.2%)	
BMI (Kg/m^2^)	Mean ± SD	28.6 ± 4.07	28.6 ± 4.57	28.3 ± 4.39	0.538
Smoking		50 (28.6%)	159 (33.8%)	137 (30.1%)	0.319

Data was presented as Mean ± SD or frequency (%).

BMI, body mass index. BMI categories were interpreted according to WHO criteria (26).

Compared with the vitamin D-sufficient and vitamin D-insufficient groups, the vitamin D-deficient group demonstrated significantly higher platelet counts, neutrophil counts, monocyte counts, NLR, PLR, MLR, SII, HbA1c, and LDL levels (all *P* < 0.05). Fasting blood glucose was significantly higher in the vitamin D-deficient group compared with the vitamin D-sufficient group. Triglyceride levels were significantly elevated in both vitamin D-insufficient and vitamin D-deficient participants, whereas HDL levels were significantly lower in the vitamin D-deficient group. No significant differences were observed for lymphocyte count, or total cholesterol (Table 2).

**Table 2:** Comparison of haematological inflammatory indices and cardiometabolic parameters according to vitamin D status.

Category	Parameter	Group 1 (Sufficient) (*n* = 175)	Group 2 (Insufficient) (*n* = 470)	Group 3 (Deficient) (*n* = 455)	*P* value
Haematological parameters	Platelets (*10^9^/L)	245.8 ± 60.29	252.1 ± 63.07	271.5 ± 76.24	**<0** **.** **001** *
		P1 = 0.544, **P2** **<** 0.001*, P3 **<** 0.001*			
	Neutrophil count (×10⁹/L)	4.38 ± 1.27	4.50 ± 1.2	4.99 ± 1.35	**<0**.001*
		P1 = 0.802, **P2** **<** 0.001*, P3 **<** 0.001*			
	Lymphocyte count (×10⁹/L)	2.01 ± 0.62	2.03 ± 0.62	1.95 ± 0.62	0.167
	Monocyte count (×10⁹/L)	0.503 ± 0.16	0.509 ± 0.17	0.537 ± 0.16	**0**.010*
		P1 = 1.00, P2 = 0.057, **P3** **=** 0.023*			
Haematological inflammatory indices	NLR	2.15 (1.67-2.88)	2.21 (1.68-2.92)	2.49 (1.87-3.53)	**<0**.001*
		P1 = 1.00, **P2** **<** 0.001*, P3 **=** 0.002*			
	PLR	118.26 (93.87-157.29)	123.745 (96.8925-160.895)	142.62 (103.54-196.24)	**0**.001*
		P1 = 1.00, **P2** **<** 0.001*, P3 **=** 0.001*			
	MLR	0.26 (0.18-0.35)	0.25 (0.18-0.3425)	0.28 (0.2-0.38)	**0**.020*
		P1 = 1.00, P2 = 0.362, **P3** **=** 0.016*			
	SII	484.83 (363.79-703.22)	552.78 (386.1275-756.2325)	670.4 (444.55-1016.58)	**<0**.001*
		P1 = 0.086, **P2** **<** 0.001*, P3 **<** 0.001*			
Glycemic profile	FBG (mg/dL)	104.6 ± 24.78	106.5 ± 24	110.2 ± 29.97	**0**.027*
		P1 = 1.00, **P2** **=** 0.049*, P3 = 0.112			
	HbA1c (%)	5.91 ± 0.92	6.01 ± 0.95	6.18 ± 1.01	**0**.003*
		P1 = 0.691, **P2** **=** 0.006*, P3 **=** 0.030*			
Lipid profile	Total cholesterol (mg/dL)	198.4 ± 37.37	198.2 ± 32.71	196.2 ± 36.61	0.637
	Triglycerides (mg/dL)	146.9 ± 59.31	161.6 ± 58.31	164.3 ± 63.48	**0**.005*
		**P1** **=** 0.019*, P2 **=** 0.004*, P3 = 1.00			
	HDL (mg/dL)	45.3 ± 10.82	44.3 ± 9.95	42.9 ± 10.77	**0**.023*
		P1 = 0.844, **P2** **=** 0.035*, P3 = 0.147			
	LDL (mg/dL)	123 ± 31.27	127.5 ± 30.39	136.5 ± 41.17	**<0**.001*
		P1 = 0.471, **P2** **<** 0.001*, P3 **<** 0.001*			

Data was presented as Mean ± SD, median (IQR), NLR, neutrophil-to-lymphocyte ratio; PLR, platelet-to-lymphocyte ratio; MLR, monocyte-to-lymphocyte ratio; SII, systemic immune-inflammation index; FBG, fasting blood glucose; HDL, high-density lipoprotein; LDL, low-density lipoprotein. Data was presented as Mean ± SD.

* statistically significant as p value <0.05, P1: p value between group 1 & 2, P2: p value between group 1 & 3, P3: p value between group 2&3. Reference clinical categories included fasting blood glucose ≥126 mg/dL for diabetes, HbA1c ≥ 6.5% for diabetes mellitus, triglycerides ≥150 mg/dL as elevated, HDL <40 mg/dL in men and <50 mg/dL in women as low, and LDL ≥130 mg/dL as elevated cardiovascular risk. Reference laboratory ranges: neutrophils 2.0–7.0 × 10⁹/L, lymphocytes 1.0–3.0 × 10⁹/L, monocytes 0.2–0.8 × 10⁹/L, and platelets 150–450 × 10⁹/L.

Correlation analyses demonstrated significant inverse associations between vitamin D levels and several inflammatory and metabolic parameters, including platelet count, neutrophil count, monocyte count, NLR, PLR, SII, fasting blood glucose, HbA1c, triglycerides, and LDL cholesterol (all *p* < 0.05). In contrast, vitamin D levels showed a weak positive correlation with HDL cholesterol (r = 0.075, *p* = 0.012). No significant correlations were observed with age, BMI, MLR, or total cholesterol.

Strong positive interrelationships were also identified among the haematological inflammatory indices. The NLR, PLR, and SII were positively correlated with glycaemic and lipid-related cardiometabolic parameters, particularly fasting blood glucose, HbA1c, and triglycerides (Table 3).

**Table 3:** Correlation between vit D level, the haematological inflammatory indices and the other parameters.

		Vit D level (ng/mL)	Haematological inflammatory indices		
			NLR	PLR	SII
Age (years)	r	0.009	0.028	0.03	0.017
	P	0.754	0.35	0.319	0.582
BMI (Kg/m^2^)	r	0.041	0.003	0.033	0.02
	P	0.912	0.912	0.270	0.511
Platelet count (×10⁹/L)	r	−0.107	0.033	0.362	0.363
	P	**<0** **.** **001** *	0.276	**<0**.001*	**<0**.001*
Neutrophil count (×10⁹/L)	r	−0.16	0.361	0.094	0.421
	P	**<0**.001*	**<0**.001*	**0**.002*	**<0**.001*
Lymphocyte count (×10⁹/L)	r	0.046	−0.557	−0.626	−0.577
	P	0.130	**<0**.001*	**<0**.001*	**<0**.001*
Monocyte count (×10⁹/L)	r	−0.061	0.06	0.057	0.087
	P	**0**.045*	**0**.046*	0.061	**0**.004*
NLR	r	−0.075	—-	0.858	0.913
	P	**0**.013*	—	**<0**.001*	**<0**.001*
PLR	r	−0.062	0.858	—-	0.919
	P	**0**.039*	**<0**.001*	—	**<0**.001*
MLR	r	−0.043	0.836	0.833	0.775
	P	0.154	**<0**.001*	**<0**.001*	**<0**.001*
SII	r	−0.111	0.913	0.919	—-
	P	**<0**.001*	**<0**.001*	**<0**.001*	—
FBG (mg/dL)	r	−0.082	0.083	0.086	0.105
	P	**0**.007*	**0**.006*	**0**.004*	**0**.001*
HbA1c (%)	r	−0.103	0.090	0.089	0.123
	P	**0**.001*	**0**.003*	**0**.003*	**<0**.001*
Total Cholesterol (mg/dL)	r	0.004	−0.033	−0.015	−0.013
	P	0.886	0.278	0.621	0.662
Triglycerides (mg/dL)	r	−0.098	0.064	0.107	0.122
	P	**0**.001*	**0**.035*	**<0**.001*	**<0**.001*
LDL (mg/dL)	r	−0.161	0.033	0.076	0.096
	P	**<0**.001*	0.268	**0**.012*	**0**.001*
HDL (mg/dL)	r	0.075	−0.008	0.009	−0.018
	P	**0**.012*	0.786	0.756	0.553

Data was presented as number.

BMI, body mass index; NLR, neutrophil-to-lymphocyte ratio; PLR, platelet-to-lymphocyte ratio; MLR, monocyte-to-lymphocyte ratio; SII, systemic immune-inflammation index; FBG, fasting blood glucose; r, correlation coefficient; HDL, high-density lipoprotein; LDL, low-density lipoprotein.

* statistically significant as p value <0.05.

In the univariate logistic regression analyses, vitamin D status was treated as the dependent outcome variable. Platelet count, neutrophil count, monocyte count, NLR, PLR, SII, fasting blood glucose, HbA1c, triglycerides, HDL, and LDL were significantly associated with vitamin D status. After multivariable adjustment, platelet count, neutrophil count, monocyte count, NLR, PLR, SII, triglycerides, and LDL remained associated with vitamin D deficiency Table 4.

**Table 4:** Univariate and multivariate logistic regression analysis for prediction of Vit D status.

Univariate logistic regression analysis			
Predictor Variable	OR	95% CI	*P* value
Age (years)	0.9964	0.9879 to 1.0050	0.411
Sex	1.0033	0.7892 to 1.2755	0.978
BMI (Kg/m^2^)	0.9847	0.9582 to 1.0118	0.265
Smoking	0.8987	0.6934 to 1.1650	0.419
Platelet count (×10⁹/L)	1.0045	1.0027 to 1.0063	**<0** **.** **001** *
Neutrophil count (×10⁹/L)	1.3827	1.2540 to 1.5246	**<0**.001*
Lymphocyte count (×10⁹/L)	0.8314	0.6844 to 1.0098	0.063
Monocyte count (×10⁹/L)	3.0878	1.4769 to 6.4556	**0**.003*
NLR	1.1105	1.0330 to 1.1938	**0**.004*
PLR	1.0018	1.0005 to 1.0032	**0**.007*
MLR	1.6968	0.9838 to 2.9264	0.057
SII	1.0006	1.0004 to 1.0009	**<0**.001*
FBG (mg/dL)	1.0059	1.0014 to 1.0104	**0**.011*
HbA1c (%)	1.2274	1.0835 to 1.3904	**0**.001*
Total cholesterol (mg/dL)	0.9983	0.9949 to 1.0018	0.343
Triglycerides (mg/dL)	1.0046	1.0028 to 1.0065	**<0**.001*
HDL (mg/dL)	0.9852	0.9739 to 0.9967	**0**.011*
LDL (mg/dL)	1.0083	1.0048 to 1.0118	**<0**.001*
Multivariate logistic regression analysis			
Platelet count (×10⁹/L)	1.0037	1.0007 to 1.0067	**0**.014*
Neutrophil count (×10⁹/L)	1.3262	1.1455 to 1.5354	**<0**.001*
Monocyte count (×10⁹/L)	2.2636	1.0565 to 4.8501	**0**.036*
NLR	0.8413	0.7177 to 0.9863	**0**.033*
PLR	0.9936	0.9900 to 0.9973	**0**.001*
SII	1.0020	1.0012 to 1.0028	**<0**.001*
FBG (mg/dL)	1.0035	0.9987 to 1.0083	0.149
HbA1c (%)	1.1207	0.9831 to 1.2775	0.088
Triglycerides (mg/dL)	1.0034	1.0014 to 1.0053	**0**.001*
HDL (mg/dL)	0.9903	0.9784 to 1.0024	0.114
LDL (mg/dL)	1.0060	1.0023 to 1.0098	**0**.001*

Data was presented as number.

OR, odds ratio; CI, confidence interval; NLR, neutrophil-to-lymphocyte ratio; PLR, platelet-to-lymphocyte ratio; MLR, monocyte-to-lymphocyte ratio; SII, systemic immune-inflammation index; HDL, high-density lipoprotein; LDL, low-density lipoprotein; NLR, neutrophil-to-lymphocyte ratio.

* statistically significant as p value <0.05.

ROC analyses demonstrated significant but clinically limited discriminatory performance. The NLR demonstrated an AUC of 0.591 (*p* < 0.001), whereas the PLR showed an AUC of 0.579 (*p* < 0.001). Similar modest discriminatory performance was observed for the MLR, SII, triglyceride, and LDL values (Supplementary Table S1, Supplementary Figure S1).

Interaction analyses were performed using adjusted multivariable logistic regression models to evaluate whether the associations between haematological inflammatory indices and vitamin D deficiency status differed across cardiometabolic risk profiles. Interaction analyses demonstrated significant associations between vitamin D status and several haematological inflammatory indices in adjusted multivariable logistic regression models. Significant interaction-term associations were observed for NLR (OR=0.9436, 95% CI: 0.9319–0.9553, *P* < 0.001), SII (OR=1.0001, 95% CI: 1.0001–1.0001, *P* < 0.001), and PLR (OR=0.9997, 95% CI: 0.9996–0.9999, *P* < 0.001), whereas the interaction term involving MLR was not statistically significant (OR=0.9824, 95% CI: 0.9312–1.0363, *P* = 0.514). These findings suggest that associations between haematological inflammatory indices and vitamin D deficiency status may vary across inflammatory profiles as shown in Table 5.

**Table 5:** Interaction terms between vitamin D status and haematological inflammatory indices in multivariable logistic regression models.

Interaction term	OR	95% CI	*P* value
vitamin D status x NLR	0.9436	0.9319 to 0.9553	<0.001*
vitamin D status x SII	1.0001	1.000003–1.00002	**<0**.001*
vitamin D status x MLR	0.9824	0.9312 to 1.0363	0.514
vitamin D status x PLR	0.9997	0.9996 to 0.9999	**<0**.001*

Models were adjusted for age, sex, BMI, and smoking status. Interaction terms were entered separately into multivariable logistic regression models. NLR, neutrophil-to-lymphocyte ratio; PLR, platelet-to-lymphocyte ratio, MLR, monocyte-to-lymphocyte ratio; SII, systemic immune-inflammation index OR, odds ratio; CI, confidence interval.

*statistically significant as p value <0.05.

## Discussion

In this cohort of 1,100 adults with comparable baseline characteristics, lower vitamin D status was primarily associated with a more pronounced pro-inflammatory haematologic profile. The most prominent differences were observed in neutrophil- and platelet-related indices, with higher NLR, PLR, and SII values among vitamin D–deficient participants, indicating a pattern of heightened systemic inflammatory activity. In contrast, some parameters, such as the MLR and total cholesterol level, did not differ significantly across vitamin D strata. Although an unfavourable cardiometabolic pattern was also observed, the haematologic inflammatory findings were the most consistent feature of the overall profile. Correlation analyses further supported this interpretation, with serum 25(OH)D showing inverse relationships with several haematological inflammatory indices and a positive relationship with HDL. Although significant, the observed correlations were relatively weak, suggesting limited clinical significance despite consistent directional associations. Thus, these haematological inflammatory indices should not be interpreted as standalone diagnostic or prognostic tools. Instead, they may serve as accessible adjunctive markers that complement established cardiovascular risk indicators, such as hs-CRP, LDL cholesterol, and traditional cardiometabolic risk scores.

The inverse associations observed between serum 25(OH)D levels and haematological inflammatory indices, particularly the NLR, PLR, and SII, are consistent with previous observational studies linking lower vitamin D status to adverse inflammatory profiles (27, 28). However, prior findings remain heterogeneous, and some studies have reported weaker or nonsignificant associations (29). In the present study, interaction analyses further indicated that relationships between inflammatory indices and cardiometabolic parameters differed by vitamin D status, particularly for the NLR and SII. These findings may indicate that inflammatory-metabolic associations vary across vitamin D categories rather than remaining uniform across all participants. However, these observations should be interpreted with caution, given the cross-sectional design, which does not permit causal inference. Furthermore, there is a possibility of residual confounding and reverse associations.

Importantly, the present findings extend beyond simple associations by suggesting that vitamin D status may be associated with differences in how inflammatory indices relate to metabolic outcomes. Composite indices such as the SII, which integrate multiple immune components, may better reflect these observed associations (30). The higher SII values observed among vitamin D–deficient individuals support the concept that Vitamin D status may be associated with differences in the broader inflammatory profile in relation to cardiometabolic risk markers. The stronger interaction observed with the SII may reflect its composite structure, which integrates multiple immune cell components and potentially provides a broader representation of systemic inflammatory activity (30, 31).

Consistent with this interpretation, vitamin D deficiency was associated with an adverse metabolic profile, including higher HbA1c, fasting glucose, triglyceride, and LDL levels, along with lower HDL levels (32). These findings align with epidemiological evidence linking vitamin D status to dyslipidaemia and impaired glycaemic control (32, 33). The observed correlations may reflect complex interrelationships among inflammatory, metabolic, and lipid-related parameters through interconnected pathways involving inflammation, insulin resistance, and lipid metabolism.

Evidence from interventional studies examining the metabolic effects of vitamin D remains inconsistent, particularly regarding glycaemic control and lipid profiles. Although some randomised controlled trials have revealed modest improvements in metabolic parameters, these effects vary across populations and are often more pronounced among individuals with baseline vitamin D deficiency (33). Meta-analyses have further indicated that supplementation may improve fasting glucose and HbA1c primarily in vitamin D-deficient populations, with less consistent benefits among those who are vitamin D sufficient (34). However, other pooled analyses have demonstrated limited or nonsignificant effects on long-term glycaemic control or the prevention of type 2 diabetes mellitus (35). This heterogeneity likely reflects differences in baseline vitamin D status, adiposity, metabolic phenotype, genetic background, and study design, as well as environmental factors such as diet and sunlight exposure. In this context, vitamin D status may represent a contextual factor associated with variation in inflammation-related metabolic associations across diverse populations. These findings should be interpreted in the context of current expert consensus on vitamin D and cardiovascular health, which emphasises personalised approaches and highlights ongoing uncertainty regarding its causal role in cardiometabolic risk. Current evidence does not support universal supplementation for cardiovascular prevention, although targeted correction of deficiencies in high-risk populations remains clinically relevant (36). Haematological inflammatory indices, such as the NLR and PLR, have been associated with adverse cardiovascular outcomes and may serve as accessible markers of systemic inflammation (37). In this study, the NLR, PLR, and SII remained associated with vitamin D status after multivariable adjustment. However, despite significance, the relatively low AUC values (approximately 0.55–0.61) indicate limited discriminatory performance. These findings suggest that the NLR, PLR, and SII are not suitable as standalone diagnostic markers for vitamin D deficiency or cardiometabolic risk prediction.

The study strengths include the relatively large sample size, comprehensive assessment of inflammatory and metabolic markers, and adjustment for key confounders. However, the cross-sectional design precludes causal inference. The use of single-time-point measurements and the lack of data on dietary intake, supplementation, and sun exposure may have contributed to residual confounding. Furthermore, we did not perform stratified subgroup analyses by diabetes status, hypertension, obesity severity, sex, or age. Because several haematological inflammatory indices are mathematically interrelated, residual multicollinearity within both regression and interaction models remains possible. This interdependence may have influenced coefficient estimates and should be considered when interpreting the observed associations. Thus, it remains unclear whether the observed associations differ across specific cardiometabolic risk phenotypes. Sex- and age-related differences in inflammatory and metabolic responses are well established and may influence the observed relationships. Future longitudinal studies should evaluate whether vitamin D-related inflammatory associations differ across clinically relevant subgroups.

Future studies should also prioritise longitudinal and interventional designs to clarify temporal relationships between vitamin D status, inflammation, and cardiometabolic risk. In particular, re-analysis of existing randomised controlled trials of vitamin D supplementation may help determine whether metabolic treatment responses differ according to baseline inflammatory index levels, thereby directly testing the interaction hypothesis suggested by the present findings.

## Conclusions

Vitamin D deficiency was associated with less favorable inflammatory and cardiometabolic profiles, including higher haematological inflammatory indices, triglycerides, LDL, fasting glucose, and HbA1c levels.

Among the evaluated markers, the NLR, PLR, and SII showed the most consistent relationships with vitamin D status. The modest discriminatory performance of these indices limits their use as standalone diagnostic tools. However, they may still offer clinical value as accessible markers within broader integrated cardiometabolic risk assessment models. These results also strengthen the view that vitamin D status should not be treated only as a background covariate.

The findings should be interpreted in light of the study’s cross-sectional design. Causality cannot be inferred. Residual confounding remains possible due to unavailable data on physical activity, dietary intake, body composition, sunlight exposure, socioeconomic factors, and vitamin D supplementation. Additionally, the study involved multiple statistical comparisons, which may have increased the risk of Type I error. Thus, the findings should be interpreted as exploratory and hypothesis-generating. Future prospective and interventional studies are warranted to clarify whether correction of vitamin D deficiency is associated with changes in inflammatory and cardiometabolic outcomes.

## Data Availability

The original contributions presented in the study are included in the article/Supplementary Material, further inquiries can be directed to the corresponding author.
