# Supplementary material for: Vitamin D status and Its association with haematological inflammatory indices and cardiometabolic risk profiles: a retrospective cross-sectional study
Source: Front Cardiovasc Med. 2026 Jun 10;13:1859225. doi: 10.3389/fcvm.2026.1859225 (PMC13290616; doi:10.3389/fcvm.2026.1859225)
Supplement: Supplementary file 1 [file Datasheet1.docx]

**Supplementary Table S1: ROC curve analysis evaluating discriminatory performance for vitamin D deficiency status**

|  | **Cutoff** | **Sensitivity** | **Specificity** | **PPV** | **NPV** | **AUC** | **P value** |
| --- | --- | --- | --- | --- | --- | --- | --- |
| **NLR** | >1.98 | 70.33 | 40.00 | 45.3 | 65.6 | 0.591 | **<0.001*** |
| **PLR** | >113.3 | 67.03 | 42.17 | 44.6 | 63.9 | 0.579 | **<0.001*** |
| **MLR** | >0.24 | 60.66 | 48.06 | 45.2 | 63.4 | 0.561 | **<0.001*** |
| **SII** | >492.09 | 70.33 | 42.79 | 46.4 | 67.2 | 0.611 | **<0.001*** |
| **Triglycerides (mg/dL)** | >144.4 | 65.49 | 42.79 | 44.7 | 63.7 | 0.579 | **<0.001*** |
| **LDL (mg/dL)** | >122.5 | 60.22 | 45.27 | 43.7 | 61.7 | 0.557 | **0.001*** |

Data was presented as frequency (%). *: statistically significant as p value <0.05. NLR: neutrophil-to-lymphocyte ratio, PLR: platelet-to-lymphocyte ratio, MLR: monocyte-to-lymphocyte ratio, SII: Systemic immune-inflammation index, HDL: high-density lipoprotein, LDL: low-density lipoprotein, PPV: positive predictive value, NPV: negative predictive value, AUC: area under the curve, *: statistically significant as p value <0.05.

| **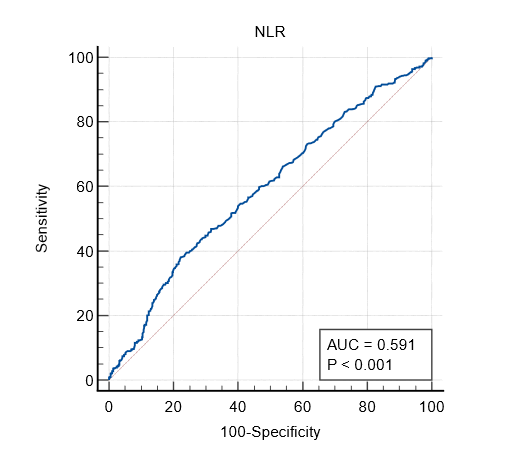**  **(A)** | **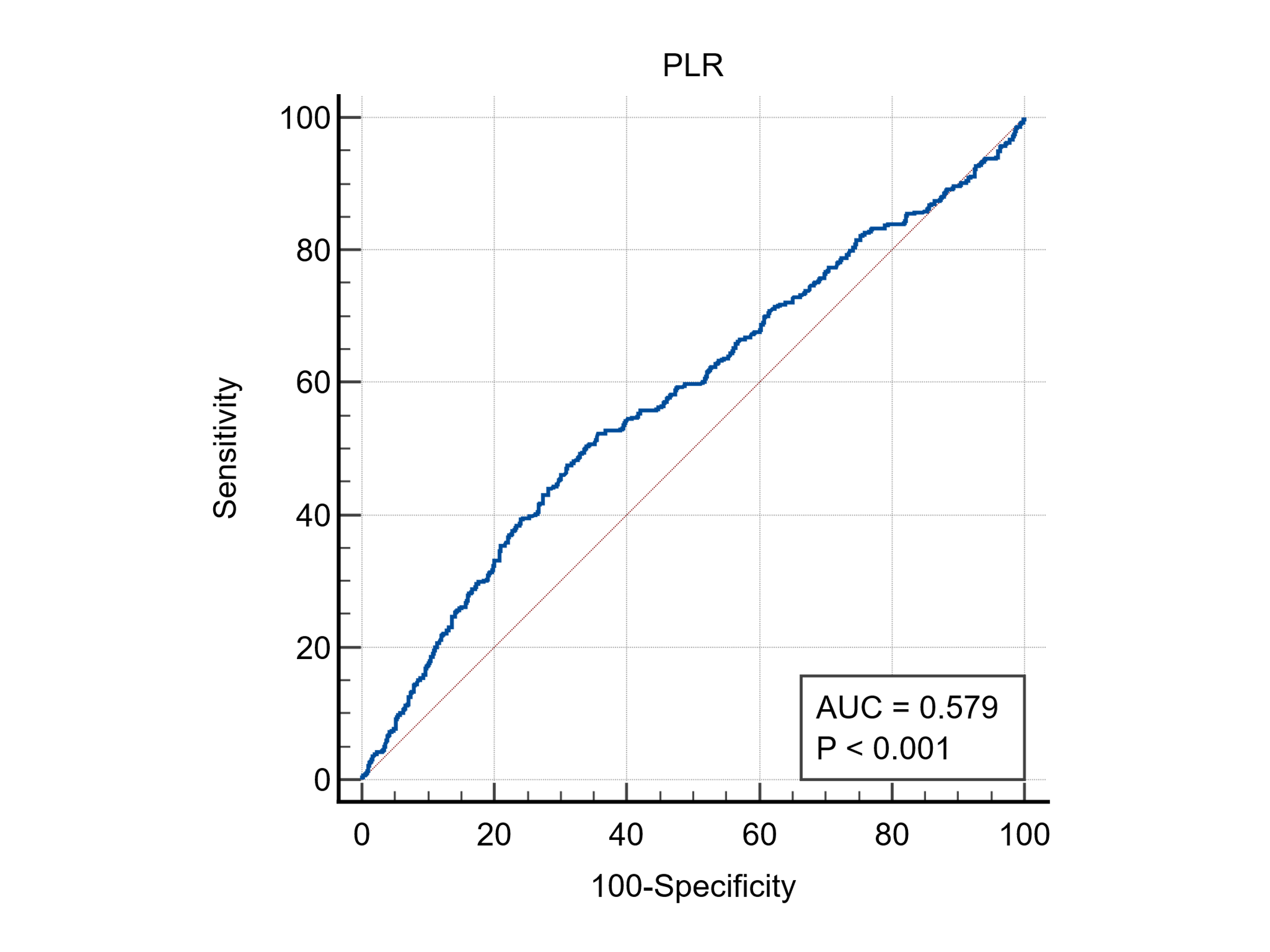**  **(B)** | **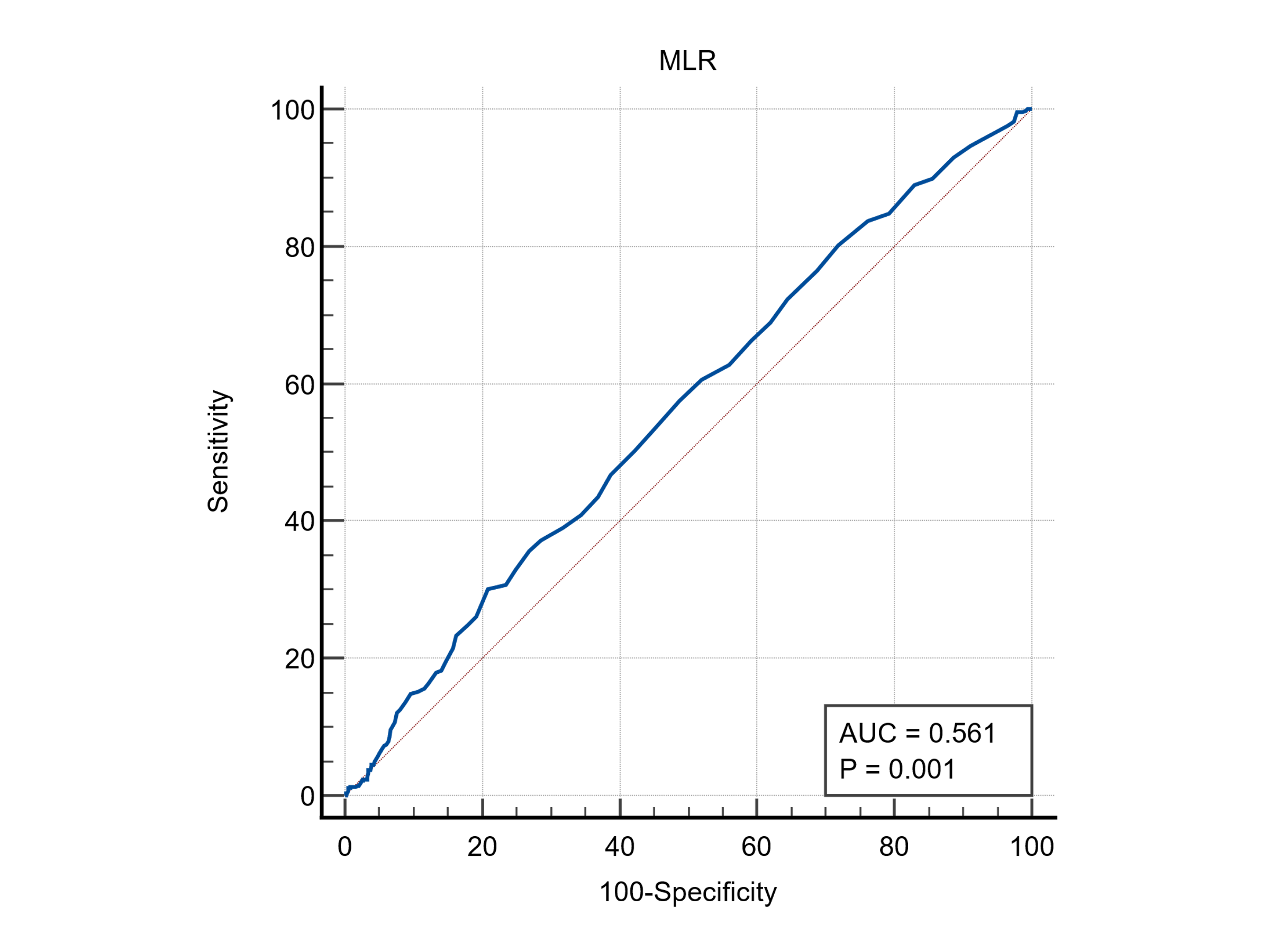**  **(C)** |
| --- | --- | --- |
| **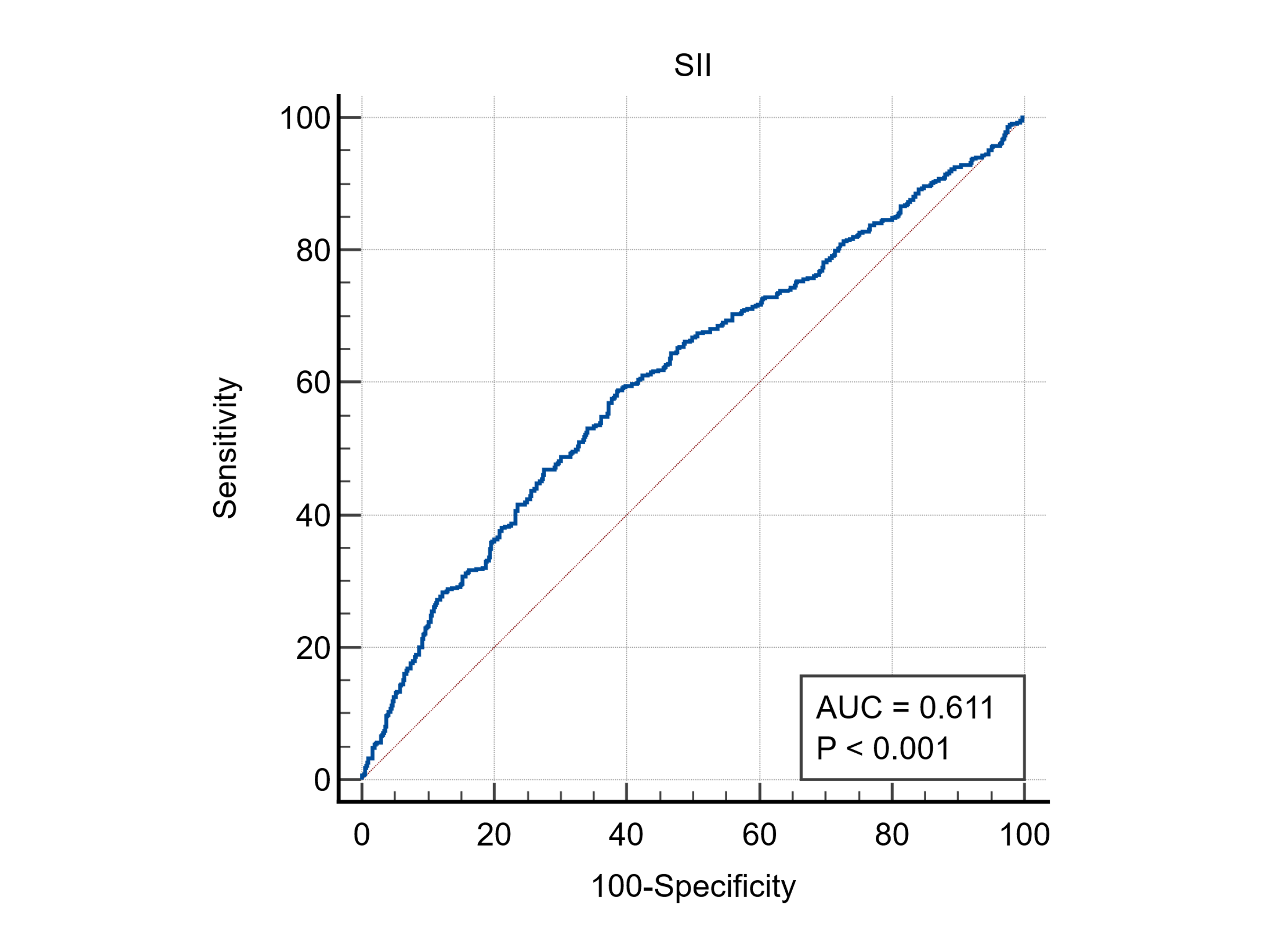**  **(D)** | **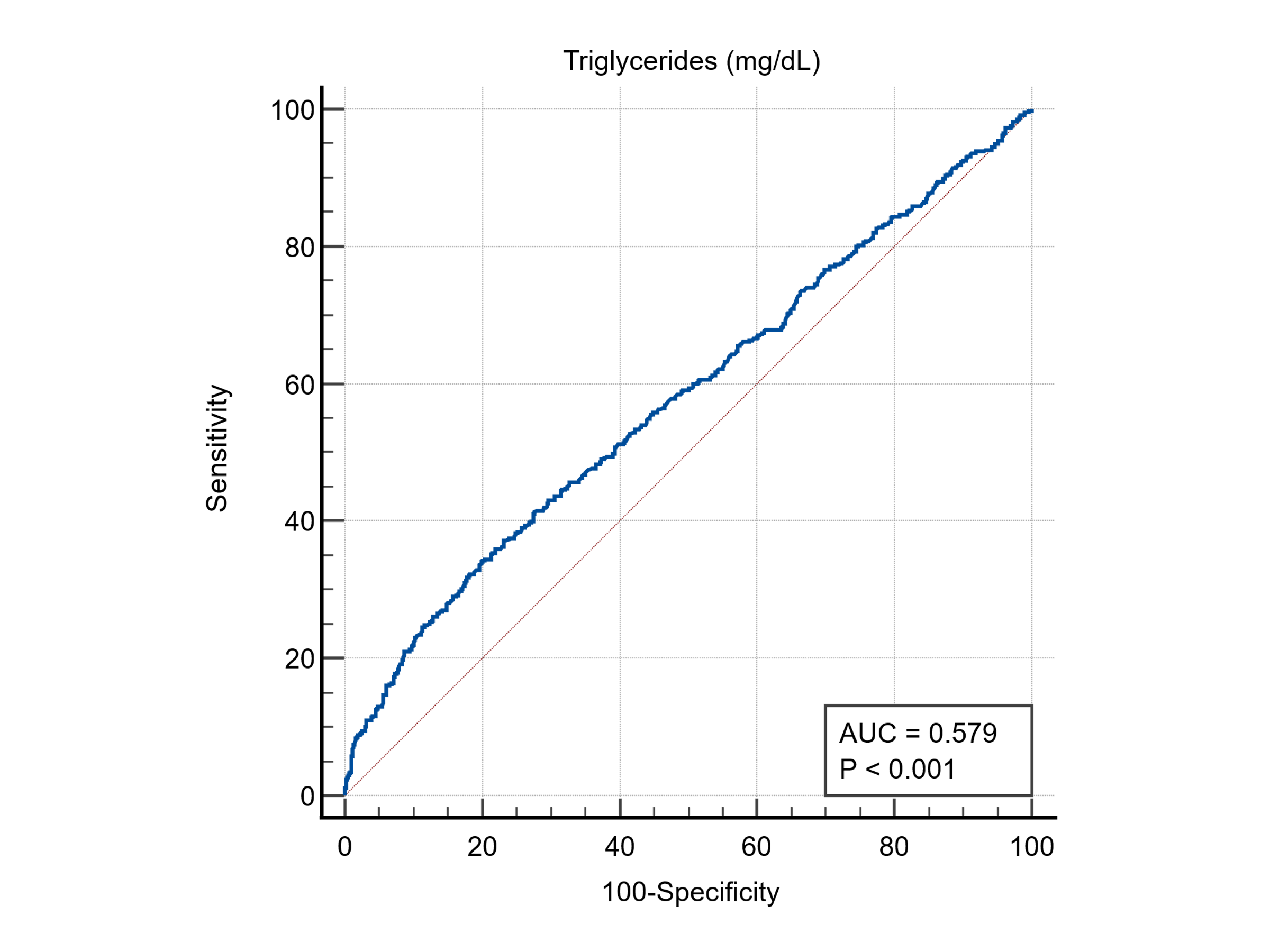**  **(E)** | **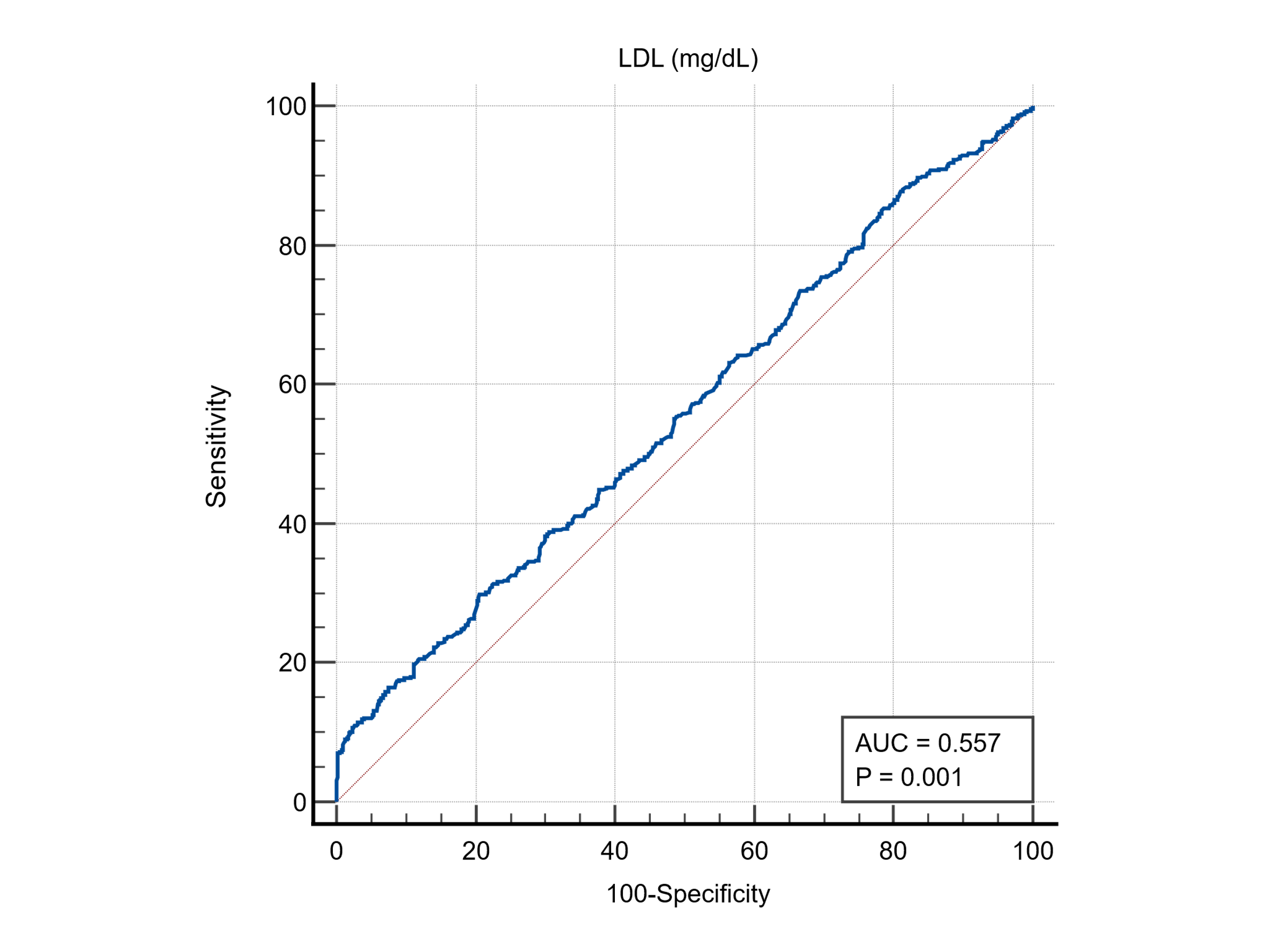**  **(F)** |

**Supplementary Figure S1: ROC curve analysis evaluating discriminatory performance for vitamin D deficiency status using (A) NLR, (B) PLR, (C) MLR, (D) SII, (E) triglycerides, and (F) LDL cholesterol.**
